# Supplementary material for: Updating the description of Rhizobium diversity associated with common bean cultivars in the Ecuadorian Andes: A phylogenetic and functional perspective
Source: PLoS One. 2026 Jan 2;21(1):e0339774. doi: 10.1371/journal.pone.0339774 (PMC12758762; doi:10.1371/journal.pone.0339774)
Supplement: S1 Table — This table provides the latitude, longitude, altitude, and soil type for each of the 46 collection sites used in this study. (DOCX) [file pone.0339774.s001.docx]

| **S1 Table.** Geographical locations and soil characteristic of the collection sites for the *Rhizobium* isolates used in this study. | | | | | | |
| --- | --- | --- | --- | --- | --- | --- |
|  | **Collection number** | **Geographical origin** | **Latitude decimal** | **Longitude decimal** | **Altitude meters** | **Soil type**^a^ |
|  |  |  |  |  |  |  |
| 1 | UCE0001 | Yaruquí/ Pichincha | -0.1699 | -78.3164 | 2580 | Andisols/ Ustivitrands |
| 2 | UCE0007 | Yaruquí/ Pichincha | -0.1699 | -78.3164 | 2580 | Andisols/ Ustivitrands |
| 3 | UCE0009 | Yaruquí/ Pichincha | -0.1699 | -78.3164 | 2580 | Andisols/ Ustivitrands |
| 4 | UCE0010 | Yaruquí/ Pichincha | -0.1699 | -78.3164 | 2580 | Andisols/ Ustivitrands |
| 5 | UCE0014 | Morochos/Imbabura | 0.2919 | -78.3151 | 2722 | Andisols/ Udivitrands |
| 6 | UCE0016 | Morochos/Imbabura | 0.2919 | -78.3151 | 2722 | Andisols/ Udivitrands |
| 7 | UCE0022 | Morochos/Imbabura | 0.2919 | -78.3151 | 2722 | Andisols/ Udivitrands |
| 8 | UCE0024 | Morochos/Imbabura | 0.2919 | -78.3151 | 2722 | Andisols/ Udivitrands |
| 9 | UCE0027 | Morochos/Imbabura | 0.2919 | -78.3151 | 2722 | Andisols/ Udivitrands |
| 10 | UCE0031 | Camuendo/Imbabura | 0.2155 | -78.2144 | 2635 | Mollisosls/ Hapludolls |
| 11 | UCE0035 | Camuendo/Imbabura | 0.2155 | -78.2144 | 2635 | Mollisosls/ Hapludolls |
| 12 | UCE0036 | Camuendo/Imbabura | 0.2155 | -78.2144 | 2635 | Mollisosls/ Hapludolls |
| 13 | UCE0042 | Camuendo/Imbabura | 0.2155 | -78.2144 | 2635 | Mollisosls/ Hapludolls |
| 14 | UCE0043 | Camuendo/Imbabura | 0.2155 | -78.2144 | 2635 | Mollisosls/ Hapludolls |
| 15 | UCE0044 | Camuendo/Imbabura | 0.2155 | -78.2144 | 2635 | Mollisosls/ Hapludolls |
| 16 | UCE0055 | Vía Caguasquí/Imbabura | 0.5009 | -78.1931 | 2314 | Mollisols/ Durustolls |
| 17 | UCE0056 | Vía Caguasquí/Imbabura | 0.5009 | -78.1931 | 2314 | Mollisols/ Durustolls |
| 18 | UCE0060 | Caguasquí/Imbabura | 0.5100 | -78.2220 | 2310 | Miscellaneous |
| 19 | UCE0075 | Alambuela/Imbabura | 0.3333 | -78.2500 | 2309 | Entisols/ Ustipsamments |
| 20 | UCE0080 | San Bartolo/Imbabura | 0.2810 | -78.3269 | 2814 | Mollisols/ Hapludolls |
| 21 | UCE0082 | San Bartolo/Imbabura | 0.2810 | -78.3269 | 2814 | Mollisols/ Hapludolls |
| 22 | UCE0085 | Quiroga/Imbabura | 0.2812 | -78.2899 | 2540 | Mollisols/ Hapludolls |
| 23 | UCE0086 | Quiroga/Imbabura | 0.2812 | -78.2899 | 2540 | Mollisols/ Hapludolls |
| 24 | UCE0117 | Turucu/Imbabura | 0.2993 | -78.2767 | 2462 | Mollisols/ Hapludolls |

**^a^** Sistema Nacional de Información de Tierras Rurales e Infraestructura Tecnológica. Ministerio de Agricultura y Ganadería,
Geoportal del Agro Ecuatoriano.

|  | **Collection**  **number** | **Geographical**  **origin** | **Latitude**  **decimal** | **Longitude**  **decimal** | **Altitude**  **meters** | **Soil type** |
| --- | --- | --- | --- | --- | --- | --- |
|  |  |  |  |  |  |  |
| 25 | UCE0119 | Turucu/Imbabura | 2.2993 | -76.2767 | 2462 | Mollisols/ Hapludolls |
| 26 | UCE0128 | Turucu/Imbabura | 0.2993 | -78.2767 | 2462 | Mollisols/ Hapludolls |
| 27 | UCE0148 | Tenta/Loja | -3.5178 | -79.2946 | 2608 | Mollisols/Haplustolls |
| 28 | UCE0150 | Tenta/Loja | -3.5178 | -79.2946 | 2608 | Mollisols/Haplustolls |
| 29 | UCE0154 | Pichig/Loja | -3.7123 | -79.2669 | 2620 | InceptisolsDystrudepts |
| 30 | UCE0154_2 | Pichig/Loja | -3.7123 | -79.2669 | 2620 | InceptisolsDystrudepts |
| 31 | UCE0154_4 | Pichig/Loja | -3.7123 | -79.2669 | 2620 | InceptisolsDystrudepts |
| 32 | UCE0155 | San Lucas/Loja | -3.7441 | -79.2667 | 2415 | Mollisols/ Hapludolls |
| 33 | UCE0156 | San Lucas/Loja | -3.7441 | -79.2667 | 2415 | Mollisols/ Hapludolls |
| 34 | UCE0157 | San Lucas/Loja | -3.7441 | -79.2667 | 2415 | Mollisols/ Hapludolls |
| 35 | UCE0158 | San Lucas/Loja | -3.7441 | -79.2667 | 2415 | Mollisols/ Hapludolls |
| 36 | UCE0158_2 | San Lucas/Loja | -3.7441 | -79.2667 | 2415 | Mollisols/ Hapludolls |
| 37 | UCE0171 | Chaltura/Imbabura | 0.3468 | -78.1815 | 2163 | Mollisols/Durustolls |
| 38 | UCE0174 | Chaltura/Imbabura | 0.3468 | -78.1815 | 2163 | Mollisols/Durustolls |
| 39 | UCE0191 | Atahualpa/Pichincha | 0.1333 | -78.3833 | 2350 | Mollisols/ Hapludolls |
| 40 | UCE0193 | Atahualpa/Pichincha | 0.1333 | -78.3833 | 2350 | Mollisols/ Hapludolls |
| 41 | UCE0197 | Atahualpa/Pichincha | 0.1333 | -78.3833 | 2350 | Mollisols/ Hapludolls |
| 42 | UCE0203 | Chatura/Imbabura | 0.3468 | -78.1815 | 2163 | Mollisols/Durustolls |
| 43 | UCE0221 | Via Pallatanga/Chimborazo | -1.9500 | -78.9700 | 2200 | Mollisols/ Hapludolls |
| 44 | UCE0224 | Via Pallatanga/Chimborazo | -2.0200 | -78.9700 | 1460 | Mollisols/ Hapludolls |
| 45 | UCE0228 | Via Pallatanga/Chimborazo | -2.0200 | -78.9700 | 1460 | Mollisols/ Hapludolls |
| 46 | UCE0231 | Salsipuedes/Chimborazo | -2.0602 | -78.9842 | 1200 | Mollisols/ Hapludolls |
