# Supplementary material for: Updating the description of Rhizobium diversity associated with common bean cultivars in the Ecuadorian Andes: A phylogenetic and functional perspective
Source: PLoS One. 2026 Jan 2;21(1):e0339774. doi: 10.1371/journal.pone.0339774 (PMC12758762; doi:10.1371/journal.pone.0339774)
Supplement: S2 Table — This table reports each isolate’s growth tolerance at the tested NaCl (1%, 2%) (w/v) concentrations and incubation temperatures (37 °C, 40°C), and lists the corresponding NCBI GenBank accession numbers for 16S rRNA, recA, glnII, and dnaK. (DOCX) [file pone.0339774.s002.docx]

**S2 Table. Growth tolerance of rhizobial isolates to NaCl and temperature, and NCBI accession numbers for *16S rRNA*,**

***recA*, and *glnII* and *dnaK* gene sequences.**

|  | **Collection number** | **Salt**  **tolerance** | | **Temperature  tolerance** | | **Accession number NCBI^a^** | | | |
| --- | --- | --- | --- | --- | --- | --- | --- | --- | --- |
|  |  | **1%** | **2%** | **37 °C** | **40 °C** | ***16S rRNA*^b^** | ***recA*** | ***glnII*** | ***dnaK*** |
| 1 | UCE0001 | - | - | - | - | PQ686550 | PQ741514 | PQ757657 | PX617437 |
| 2 | UCE 0007 | - | - | - | - | - | PQ741518 | - | - |
| 3 | UCE0009 | - | - | +++ | ++ | - | PQ741520 | - | - |
| 4 | UCE0010 | - | - | - | - | - | PQ741521 | - | - |
| 5 | UCE0014 | +++ | +++ | +++ | - | - | PQ741522 | - | - |
| 6 | UCE0016 | - | - | - | - | - | PQ741524 | - | - |
| 7 | UCE0022 | - | - | - | - | - | PQ741523 | - | - |
| 8 | UCE0024 | - | - | - | - | PQ686551 | PQ741527 | PQ757658 | PX617448 |
| 9 | UCE0027 | - | - | - | - | PQ686552 | PQ741529 | PQ757660 | PX617449 |
| 10 | UCE0031 | - | - | - | - | - | PQ741532 | - | - |
| 11 | UCE0035 | - | - | - | - | - | PQ741533 | - | - |
| 12 | UCE0036 | - | - | - | - | - | PQ741534 | - | - |
| 13 | UCE0042 | - | - | - | - | PQ686553 | PQ741535 | PQ757661 | PX617447 |
| 14 | UCE0043 | - | - | - | - | PQ686554 | PQ741536 | PQ757662 | PX617438 |
| 15 | UCE0044 | - | - | - | - | - | PQ741537 | - | - |
| 16 | UCE0055 | - | - | +++ | +++ | PQ686556 | PQ741542 | PQ757664 | PX617453 |
| 17 | UCE0056 | - | - | - | - | - | PQ741543 | - | - |
| 18 | UCE0060 | - | - | - | - | PQ686557 | PQ741544 | PQ757665 | PX617445 |
| 19 | UCE0075 | - | - | - | - | - | PQ741548 | PQ757667 | PX617455 |
| 20 | UCE0080 | - | - | +++ | - | PQ686560 | PQ741551 | PQ757669 | PX617443 |
| 21 | UCE0082 | - | - | ++ | - | PQ686551 | PQ741552 | PQ757670 | PX617440 |
| 22 | UCE0085 | - | - | - | - | - | PQ741554 | - | - |
| 23 | UCE0086 | - | - | - | - | - | PQ741555 | - | - |
| 24 | UCE0117 | - | - | - | - | - | PQ741557 | PQ757671 | PX617436 |
| **^a^** The National Center for Biotechnology Information (NCBI). **^b^** The 16S rRNA gene was sequenced from the isolates to confirm their genus, and the sequences were deposited in the NCBI database. Growth tolerance code: (+++) = high growth; (++) = moderate growth; (-) = no growth. | | | | | | | | |  |

|  | **Collection number** | **Salt**  **tolerance** | | **Temperature tolerance** | | **Accession number NCBI** | | | |
| --- | --- | --- | --- | --- | --- | --- | --- | --- | --- |
|  |  | **1%** | **2%** | **37 °C** | **40 °C** | ***16S rRNA*** | ***recA*** | ***glnII*** | ***dnak*** |
| 25 | UCE0119 | - | - | - | - | - | PQ741559 | - | - |
| 26 | UCE0128 | - | - | - | - | - | PQ741563 | - | - |
| 27 | UCE0148 | - | - | +++ | ++ | PQ686562 | PQ741566 | PQ757672 | PX617441 |
| 28 | UCE0150 | +++ | ++ | - | - | PQ686563 | PQ741568 | PQ757673 | PX617456 |
| 29 | UCE0154 | - | - | - | - | - | PQ741569 | - | - |
| 30 | UCE0154_2 | - | - | - | - | - | PQ741571 | - | - |
| 31 | UCE0154_4 | ++ | - | ++ | - | - | PQ741573 | - | - |
| 32 | UCE0155 | - | - | - | - | - | PQ741574 | - | - |
| 33 | UCE0156 | - | - | - | - | - | PQ741575 | - | - |
| 34 | UCE0157 | +++ | ++ | +++ | - | - | PQ741576 | - | - |
| 35 | UCE0158 | - | - | - | - | PQ686564 | PQ741577 | PQ757674 | PX617457 |
| 36 | UCE0158_2 | - | - | - | - | PQ686565 | PQ741578 | PQ757675 | PX617458 |
| 37 | UCE0171 | - | - | +++ | - | - | PQ741581 | - | - |
| 38 | UCE0174 | ++ | - | +++ | +++ | - | PQ741582 | - | - |
| 39 | UCE0191 | - | - | - | - | - | PQ741584 | - | - |
| 40 | UCE0193 | - | - | - | - | - | PQ741585 | - | - |
| 41 | UCE0197 | - | - | - | - | - | PQ741587 | - | - |
| 42 | UCE0203 | - | - | - | - | PQ686566 | PQ741590 | PQ757676 | PX6174144 |
| 43 | UCE0221 | - | - | - | - | - | PQ741591 | - | - |
| 44 | UCE0224 | ++ | - | +++ | - | - | PQ741592 | PQ757677 | PX617450 |
| 45 | UCE0228 | ++ | - | - | - | PQ686567 | PQ741594 | PQ757678 | PX617451 |
| 46 | UCE0231 | - | - | +++ | ++ | PQ686568 | PQ741595 | PQ768511 | PX617452 |
