# Supplementary material for: Updating the description of Rhizobium diversity associated with common bean cultivars in the Ecuadorian Andes: A phylogenetic and functional perspective
Source: PLoS One. 2026 Jan 2;21(1):e0339774. doi: 10.1371/journal.pone.0339774 (PMC12758762; doi:10.1371/journal.pone.0339774)
Supplement: S3 Table — Lists primer names and sequences, reagent concentrations/volumes per PCR reaction, and the cycling parameters used for each target gene. (DOCX) [file pone.0339774.s003.docx]

| **S3 Table.** Primer sequences, PCR Reaction components, and thermal cycling parameters used for amplification of target genes. | | | | | |
| --- | --- | --- | --- | --- | --- |
| **Gene** | **Primer sequence 5'-3'** | **PCR reaction mix** | **Cycling parameters** | **Reference** | **Amplicon size bp** |
|  |  |  |  |  |  |
| *16S rRNA* | 16S_rRnaU_F:  AGAGTTTGATCCTGGCTCAG  16S_rRnaU_R:  ACGGATACCTTGTTACGACTT | 25 µL PCR: Thermo Scientific DreamTaq Green PCR Master Mix (2X) 25 µM 16S_rRnaU_F primer,  25 µM 16S_rRnaU_R primer,  2 µL DNA*, PCR water and 4% DMSO. | Modified for this study:  94 °C for 3 min;  **20x** (94 °C for 10 s, 62 °C for 30 s, 72 °C for 90 s );  **15x** (94 °C for 10 s, 60 °C for 30 s, 72 °C for 90 s ); 72 for 5 min. | [1] | ~867 |
|  |  |  |  |  |  |
|  |  |  |  |  |  |
|  |  |  |  |  |  |
| *recA** | recA_F: ATCGAGCGGTCGTTCGGCAAGGG  recA_R:  TTGCGCAGCGCCTGGCTCAT | 25 µL PCR: Thermo Scientific DreamTaq Green PCR Master Mix (2X) 10 µM recA_F primer,  10 µM recA_R primer,  20 ng/µL DNA*, PCR water and 4% DMSO. | Modified for this study: 95 °C for 3 min;  **30x** (95 °C for 30 s, 67 °C for 30 s, 72 °C for 1 min);  72 for 5 min. | [2] | ~404 |
|  |  |  |  |  |  |
|  |  |  |  |  |  |
| *glnII* | glnII_12F:  AAGCTCGAGTACATYTGGCTCGACGG  glnII_R:  SGAGCCGTTCCAGTCGGTGTCG | 25 µL PCR:  Thermo Scientific DreamTaq Green PCR Master Mix (2X),  10 µM glnII_12F primer,  10 µM glnII_R primer,  20 ng/µL DNA, PCR water and 4% DMSO. | Modified for this study:  94 °C for 3 min;  **20x** (94 °C for 10 s, 62 °C for 30 s, 72 °C for 90 s );  **15x** (94 °C for 10 s, 60 °C for 30 s, 72 °C for 90 s ); 72 for 5 min. | [3] | ~647 |
|  |  |  |  |  |  |
|  |  |  |  |  |  |
|  |  |  |  |  |  |
|  |  |  |  |  |  |
| *dnaK* | dnaK_1468F: AAGGAGCAGCAGATCCGCATCCA  DnaK_1772R: GTACATGGCCTCGCCGAGCTTCA | 25 µL PCR:  Thermo Scientific DreamTaq Green PCR Master Mix (2X)  10 µm dnaK_1468F primer,  10 µM dnaK_1772R primer,  20 ng/µL DNA, PCR water and 4%  DMSO. | Modified for this study:  95 °C for 3 min;  **30x** (95 °C for 30 s, 62 °C for 30 s, 72 °C for 1 min); 72 for 5 min. | [4] | ~304 |
