# Supplementary material for: Updating the description of Rhizobium diversity associated with common bean cultivars in the Ecuadorian Andes: A phylogenetic and functional perspective
Source: PLoS One. 2026 Jan 2;21(1):e0339774. doi: 10.1371/journal.pone.0339774 (PMC12758762; doi:10.1371/journal.pone.0339774)
Supplement: S4 Table — For each isolate–variety combination, the table reports the mean number of nodules (µ) and corresponding standard deviation (σ), based on three independent biological replicates. Nodules were scored 45 days post–inoculation. Isolates from Chimborazo were excluded due to unavailability at the moment of the greenhouse trial. In addition, an Excel (.xlsx) file will be included containing the mean (µ) and standard deviation (σ) values for each of the three independent biological replicates for the following variables: count of nodules > 2 mm, number of leghemoglobin–positive nodules, and percentage (%) of leghemoglobin-positive nodules. In yellow, isolates molecularly characterized with three housekeeping genes recA, glnII and dnaK. (DOCX) [file pone.0339774.s004.docx]

**S4 Table. Nodulation performance of two common bean (Phaseolus vulgaris) varieties (Canario [Ca] and Centenario [Ce]) inoculated with 43 rhizobial isolates and two reference strains under greenhouse conditions.** For each isolate–variety combination, the table reports the mean number of nodules (µ) and corresponding standard deviation (σ), based on three independent biological replicates. Nodules were scored 45 days post–inoculation. Isolates from Chimborazo were excluded due to unavailability at the moment of the greenhouse trial. In addition, an Excel (.xlsx) file will be included containing the mean (µ) and standard deviation (σ) values for each of the three independent biological replicates for the following variables: count of nodules > 2 mm, number of leghemoglobin–positive nodules, and percentage (%) of leghemoglobin-positive nodules. In yellow, isolates molecularly characterized with three housekeeping genes *recA*, *glnII* and *dnaK*.

|  | **Isolate  Collection  Number** | **Geographical**  **origin** | **Bean variety Canario  (Ca)** | | **Bean variety Centenario**  **(Ce)** | |
| --- | --- | --- | --- | --- | --- | --- |
|  |  |  | $\bar{x}$ | Sd | $\bar{x}$ | Sd |
| 1 | **UCE**0001 | Pichincha | 11.7 | 3.1 | 82.2 | 6.7 |
| 2 | **UCE**0007 | Pichincha | 149.0 | 23.6 | 40.7 | 15.3 |
| 3 | **UCE**0009 | Pichincha | 60.3 | 67.3 | 83.0 | 24.9 |
| 4 | **UCE**0010 | Pichincha | 29.3 | 4.2 | 32.3 | 7.5 |
| 5 | **UCE**0014 | Imbabura | 4.3 | 4.9 | 12.3 | 12.7 |
| 6 | **UCE**0016 | Imbabura | 75.0 | 26.7 | 22.3 | 11.6 |
| 7 | **UCE**0022 | Imbabura | 121.3 | 35.3 | 101.0 | 68.1 |
| 8 | **UCE**0024 | Imbabura | 69.0 | 19.1 | 16.7 | 4.1 |
| 9 | **UCE**0027 | Imbabura | 1.3 | 0.6 | 88.7 | 22.5 |
| 10 | **UCE**0031 | Imbabura | 16.3 | 7.8 | 17.0 | 6.6 |
| 11 | **UCE**0035 | Imbabura | 4.3 | 3.1 | 65.7 | 53.0 |
| 12 | **UCE**0036 | Imbabura | 21.3 | 2.1 | 43.3 | 16.5 |
| 13 | **UCE**0042 | Imbabura | 1.6 | 1.2 | 15.7 | 16.8 |
| 14 | **UCE**0043 | Imbabura | 57.0 | 12.5 | 11.3 | 11.1 |
| 15 | **UCE**0044 | Imbabura | 5.0 | 6.1 | 58.7 | 59.0 |
| 16 | **UCE**0055 | Imbabura | 17.3 | 4.7 | 1.0 | 0.0 |
| 17 | **UCE**0056 | Imbabura | 7.3 | 2.1 | 19.0 | 5.3 |
| 18 | **UCE**0060 | Imbabura | 17.7 | 9.3 | 17.7 | 9.3 |
| 19 | **UCE**0075 | Imbabura | 6.0 | 5.3 | 58.3 | 7.4 |
| 20 | **UCE**0080 | Imbabura | 5.7 | 5.0 | 36.3 | 12.3 |
| 21 | **UCE**0082 | Imbabura | 100.7 | 10.6 | 70.3 | 2.5 |
| 22 | **UCE**0085 | Imbabura | 14.0 | 7.8 | 25.0 | 7.0 |
| 23 | **UCE**0086 | Imbabura | 94.0 | 24.6 | 84.3 | 12.1 |
| 24 | **UCE**0117 | Imbabura | 33.7 | 9.3 | 67.7 | 10.3 |
| 25 | **UCE**0119 | Imbabura | 142.3 | 18.7 | 175.7 | 31.6 |
| 26 | **UCE**0128 | Imbabura | 58.7 | 7.2 | 4.7 | 6.4 |
| 27 | **UCE**0148 | Loja | NA | NA | NA | NA |
| 28 | **UCE**0150 | Loja | 38.0 | 18.1 | 24.0 | 14.1 |
| 29 | **UCE**0154 | Loja | 143.7 | 29.4 | 40.3 | 15.5 |
| 30 | **UCE**0154.2 | Loja | 87.7 | 11.2 | 85.3 | 8.5 |
| 31 | **UCE**0154.4 | Loja | 85.0 | 8.5 | 49.0 | 47.3 |
| 32 | **UCE**0155 | Loja | 143.3 | 19.4 | 125.3 | 108.3 |
| 33 | **UCE**0156 | Loja | 174.3 | 16.8 | 51.3 | 49.1 |
| 34 | **UCE**0157 | Loja | 15.0 | 8.0 | 4.3 | 3.5 |
| 35 | **UCE**0158 | Loja | 79.0 | 14.5 | 7.7 | 6.1 |
| 36 | **UCE**0158.2 | Loja | 9.0 | 7.0 | 131.3 | 41.0 |
| 37 | **UCE**0171 | Imbabura | 75.7 | 13.7 | 20.7 | 5.9 |
| 38 | **UCE**0174 | Imbabura | 46.3 | 15.0 | 24.3 | 17.9 |
| 39 | **UCE**0191 | Pichincha | 6.3 | 4.7 | 44.0 | 14.1 |
| 40 | **UCE**0193 | Pichincha | 25.7 | 7.4 | 26.3 | 5.9 |
| 41 | **UCE**0197 | Pichincha | 54.0 | 21.1 | 225.7 | 43.1 |
| 42 | **UCE**0203 | Imbabura | 5.7 | 8.1 | 31.3 | 41.0 |
| 43 | **UMR**1632* | USA | 30.3 | 11.0 | 12.0 | 11.0 |
| 44 | **UMR**1899* | USA | 33.3 | 11.1 | 127.7 | 34.6 |

* UMR1899 (*Rhizobium* *tropici* IIB CIAT 899^T^) and UMR1632 (*Rhizobium etli* CIAT 632).
