# Supplementary material for: Updating the description of Rhizobium diversity associated with common bean cultivars in the Ecuadorian Andes: A phylogenetic and functional perspective
Source: PLoS One. 2026 Jan 2;21(1):e0339774. doi: 10.1371/journal.pone.0339774 (PMC12758762; doi:10.1371/journal.pone.0339774)
Supplement: S5 Table — Raw data from the three replications of the three independent biological replicates for the following variables: i) number of nodules, ii) count of nodules > 2 mm, iii) number of leghemoglobin–positive nodules, and iv) percentage (%) of leghemoglobin-positive nodules. (DOCX) [file pone.0339774.s005.docx]

**S5 Table. Raw data of the quantitative variables of nodulation performance evaluated in two varieties of bush bean Centenario (Ce) and Canario (Ca) after being inoculated with rhizobia isolates.** Raw data from the three replications of the three independent biological replicates for the following variables: i) number of nodules, ii) count of nodules > 2 mm, iii) number of leghemoglobin–positive nodules, and iv) percentage (%) of leghemoglobin-positive nodules.

| **Bean  Variety** | **Replication** | **Isolate  Collection Number** | **Total nodules count  per plant** | **Total nodules count  per plant > 2 mm** | **Number of leghemoglobin–positive  nodules** | **Percentage (%) of  leghemoglobin‑positive nodules** |
| --- | --- | --- | --- | --- | --- | --- |
|  |  |  |  |  |  |  |
|  |  |  |  |  |  |  |
| Ce | 1 | UCE0001 | 86 | 56 | 42 | 75 |
| Ce | 2 | UCE0001 | 87 | 45 | 45 | 100 |
| Ce | 3 | UCE0001 | 75 | 60 | 59 | 98 |
| Ce | 1 | UCE0024 | 16 | 15 | 15 | 100 |
| Ce | 2 | UCE0024 | 21 | 21 | 10 | 48 |
| Ce | 3 | UCE0024 | 13 | 13 | 10 | 77 |
| Ce | 1 | UCE0027 | 98 | 76 | 70 | 92 |
| Ce | 2 | UCE0027 | 105 | 100 | 94 | 94 |
| Ce | 3 | UCE0027 | 63 | 52 | 48 | 92 |
| Ce | 1 | UCE0042 | 1 | 1 | 0 | 0 |
| Ce | 2 | UCE0042 | 12 | 5 | 5 | 100 |
| Ce | 3 | UCE0042 | 34 | 27 | 15 | 56 |
| Ce | 1 | UCE0043 | 1 | 1 | 1 | 100 |
| Ce | 2 | UCE0043 | 23 | 2 | 2 | 100 |
| Ce | 3 | UCE0043 | 10 | 1 | 1 | 100 |
| Ce | 1 | UCE0055 | 1 | 1 | 1 | 100 |
| Ce | 2 | UCE0055 | 1 | 1 | 1 | 100 |
| Ce | 3 | UCE0055 | 1 | 1 | 1 | 100 |
| Ce | 1 | UCE0060 | 99 | 85 | 85 | 100 |
| Ce | 2 | UCE0060 | 145 | 120 | 100 | 83 |
| Ce | 3 | UCE0060 | 80 | 73 | 73 | 100 |
| **Bean  Variety** | **Replication** | **Isolate  Collection Number** | **Total nodules count  per plant** | **Total nodules count  per plant > 2mm** | **Number of leghemoglobin–positive  nodules** | **Percentage (%) of  leghemoglobin‑positive nodules** |
| Ce | 1 | UCE0080 | 33 | 5 | 1 | 20 |
| Ce | 2 | UCE0080 | 50 | 3 | 2 | 67 |
| Ce | 3 | UCE0080 | 26 | 2 | 2 | 100 |
| Ce | 1 | UCE0082 | 73 | 65 | 61 | 94 |
| Ce | 2 | UCE0082 | 70 | 50 | 49 | 98 |
| Ce | 3 | UCE0082 | 68 | 68 | 60 | 88 |
| Ce | 1 | UCE0117 | 79 | 67 | 66 | 99 |
| Ce | 2 | UCE0117 | 65 | 56 | 53 | 95 |
| Ce | 3 | UCE0117 | 59 | 57 | 53 | 93 |
| Ce | 1 | UCE0150 | 11 | 11 | 11 | 100 |
| Ce | 2 | UCE0150 | 22 | 12 | 12 | 100 |
| Ce | 3 | UCE0150 | 39 | 30 | 26 | 87 |
| Ce | 1 | UCE0158 | 1 | 1 | 1 | 100 |
| Ce | 2 | UCE0158 | 9 | 2 | 1 | 50 |
| Ce | 3 | UCE0158 | 13 | 1 | 1 | 100 |
| Ce | 1 | UCE0158.2 | 50 | 48 | 45 | 94 |
| Ce | 1 | UCE0203 | 1 | 1 | 0 | 0 |
| Ce | 2 | UCE0203 | 15 | 10 | 5 | 50 |
| Ce | 3 | UCE0203 | 78 | 65 | 61 | 94 |
| Ca | 1 | UCE0001 | 11 | 2 | 1 | 50 |
| Ca | 2 | UCE0001 | 15 | 2 | 2 | 100 |
| Ca | 3 | UCE0001 | 9 | 2 | 1 | 50 |
| Ca | 1 | UCE0024 | 51 | 45 | 45 | 100 |
| Ca | 2 | UCE0024 | 67 | 23 | 23 | 100 |
| Ca | 3 | UCE0024 | 89 | 22 | 19 | 86 |
| Ca | 1 | UCE0027 | 1 | 1 | 1 | 100 |
| Ca | 2 | UCE0027 | 1 | 1 | 1 | 100 |
| **Bean  Variety** | **Replication** | **Isolate  Collection Number** | **Total nodules count  per plant** | **Total nodules count  per plant > 2 mm** | **Number of leghemoglobin–positive  nodules** | **Percentage (%) of  leghemoglobin‑positive nodules** |
| Ca | 3 | UCE0027 | 2 | 2 | 2 | 100 |
| Ca | 1 | UCE0042 | 1 | 1 | 1 | 100 |
| Ca | 2 | UCE0042 | 3 | 3 | 2 | 67 |
| Ca | 3 | UCE0042 | 1 | 1 | 1 | 100 |
| Ca | 1 | UCE0043 | 70 | 53 | 53 | 100 |
| Ca | 2 | UCE0043 | 45 | 34 | 34 | 100 |
| Ca | 3 | UCE0043 | 56 | 23 | 23 | 100 |
| Ca | 1 | UCE0055 | 12 | 12 | 12 | 100 |
| Ca | 2 | UCE0055 | 19 | 1 | 1 | 100 |
| Ca | 3 | UCE0055 | 21 | 6 | 6 | 100 |
| Ca | 1 | UCE0060 | 15 | 4 | 4 | 100 |
| Ca | 2 | UCE0060 | 28 | 1 | 1 | 100 |
| Ca | 3 | UCE0060 | 10 | 3 | 3 | 100 |
| Ca | 1 | UCE0080 | 1 | 1 | 1 | 100 |
| Ca | 2 | UCE0080 | 11 | 2 | 1 | 50 |
| Ca | 3 | UCE0080 | 5 | 3 | 1 | 33 |
| Ca | 1 | UCE0082 | 91 | 75 | 75 | 100 |
| Ca | 2 | UCE0082 | 112 | 87 | 87 | 100 |
| Ca | 3 | UCE0082 | 99 | 69 | 69 | 100 |
| Ca | 1 | UCE0117 | 40 | 37 | 37 | 100 |
| Ca | 2 | UCE0117 | 38 | 38 | 38 | 100 |
| Ca | 3 | UCE0117 | 23 | 12 | 12 | 100 |
| Ca | 1 | UCE0150 | 21 | 1 | 1 | 100 |
| Ca | 2 | UCE0150 | 36 | 2 | 2 | 100 |
| Ca | 3 | UCE0150 | 57 | 1 | 1 | 100 |
| Ca | 1 | UCE0158 | 94 | 90 | 90 | 100 |
| **Bean  Variety** | **Replication** | **Isolate  Collection Number** | **Total nodules count  per plant** | **Total nodules count  per plant > 2 mm** | **Number of leghemoglobin–positive  nodules** | **Percentage (%) of  leghemoglobin‑positive nodules** |
| Ca | 1 | UCE0158.2 | 1 | 1 | 1 | 100 |
| Ca | 2 | UCE0158.2 | 12 | 2 | 1 | 50 |
| Ca | 3 | UCE0158.2 | 14 | 2 | 1 | 50 |
| Ca | 1 | UCE0203 | 1 | 1 | 1 | 100 |
| Ca | 2 | UCE0203 | 15 | 1 | 1 | 100 |
| Ca | 3 | UCE0203 | 1 | 1 | 1 | 100 |
