# Supplementary figures and images for: Updating the description of Rhizobium diversity associated with common bean cultivars in the Ecuadorian Andes: A phylogenetic and functional perspective
Source: PLoS One. 2026 Jan 2;21(1):e0339774. doi: 10.1371/journal.pone.0339774 (PMC12758762; doi:10.1371/journal.pone.0339774)

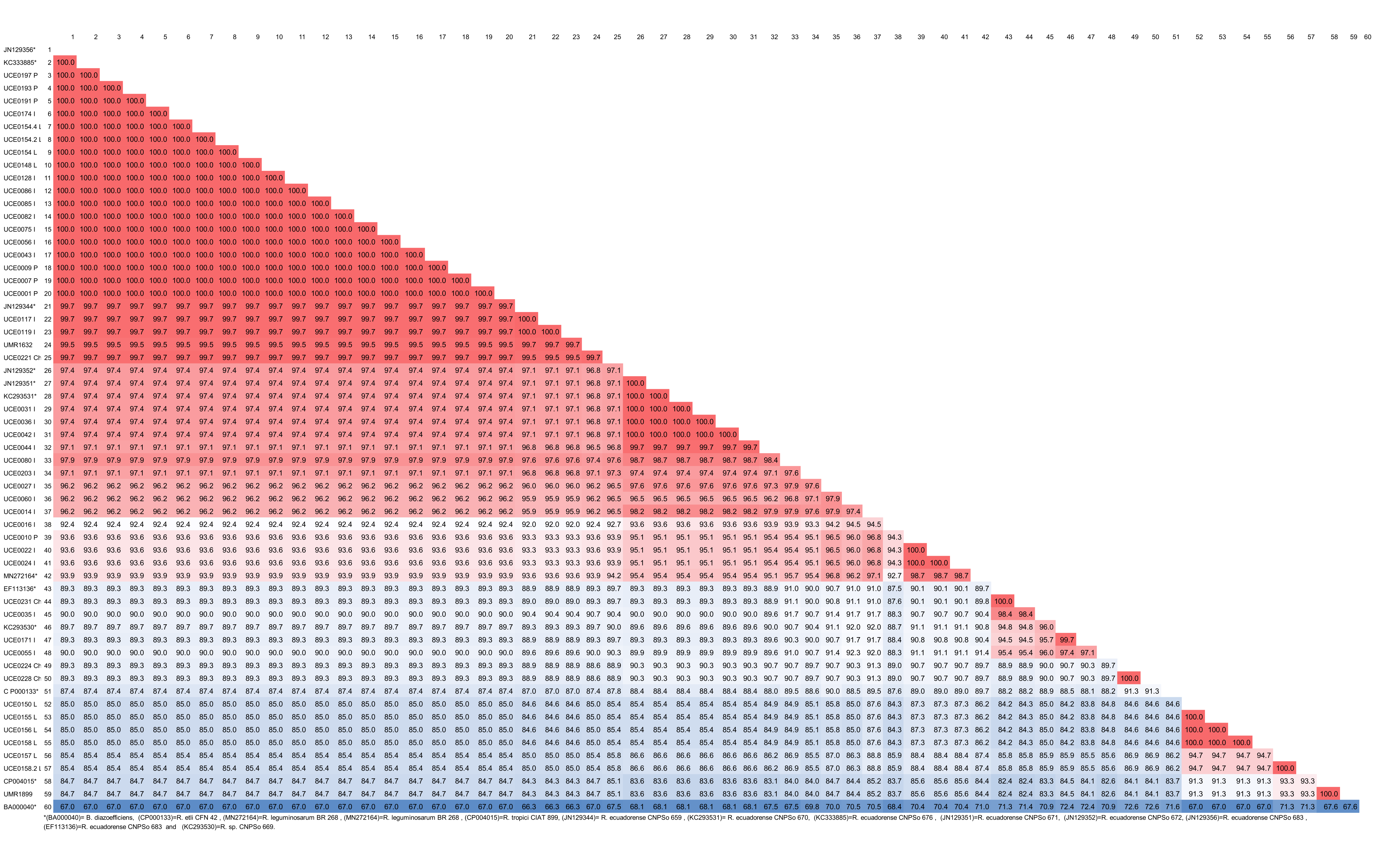

Supplement: S1 Fig — Pairwise percent–identity values estimates were calculated from a 395 bp alignment of recA sequences to quantify the evolutionary divergence between each study isolate and its nearest described type strain. Evolutionary analyses were carried out using MEGA X; alignment and analysis parameters are detailed in the Methods. (TIFF) [file pone.0339774.s006.tiff]

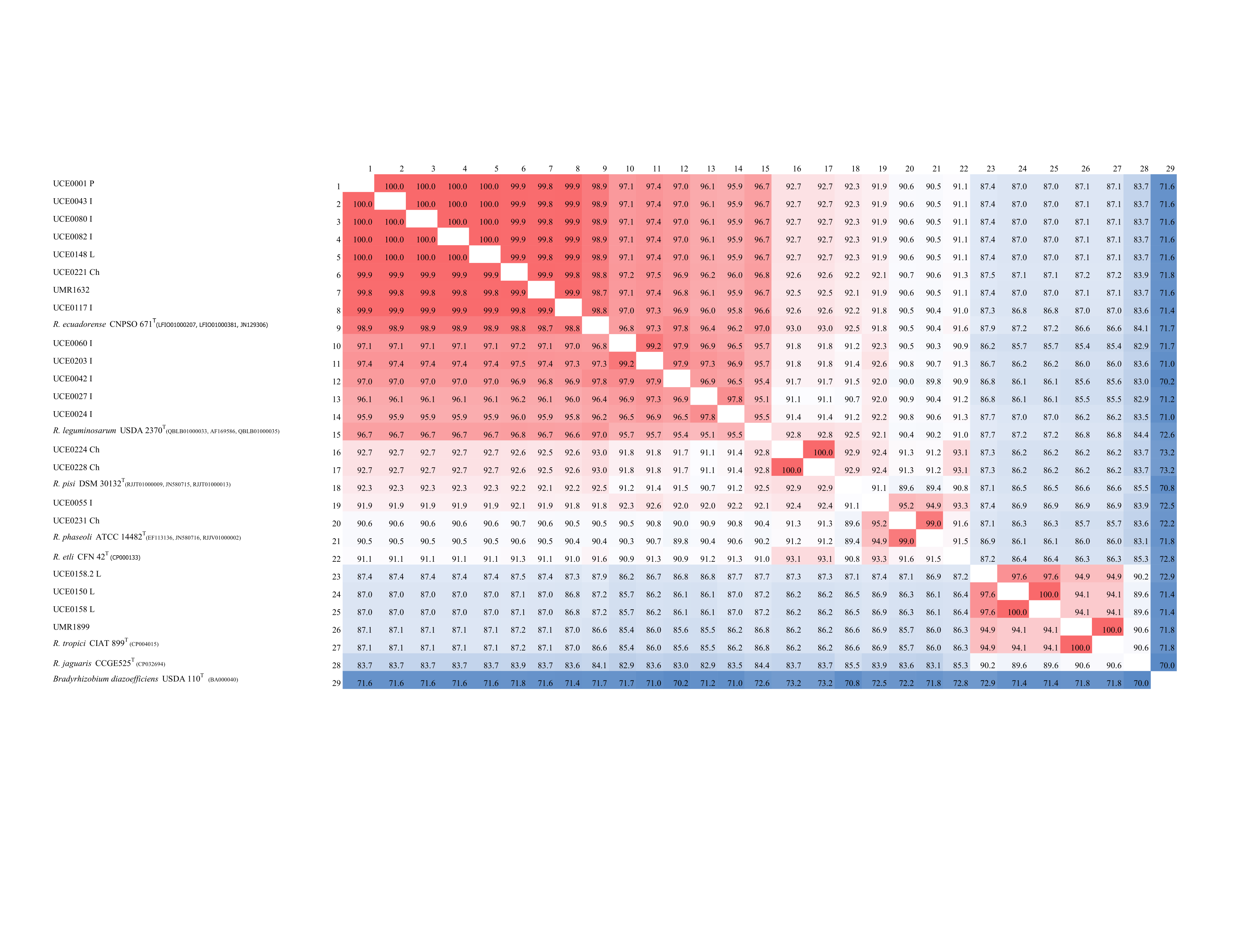

Supplement: S2 Fig — Pairwise percent–identity estimates were calculated from a 1,115 bp concatenated alignment (recA, glnII, dnaK) to quantify evolutionary divergence between each study isolate and its nearest described type strain. Evolutionary analyses were performed using MEGA X software; alignment and analysis parameters are described in the Methods. (TIF) [file pone.0339774.s007.tif]
