# Supplementary material for: Updating the description of Rhizobium diversity associated with common bean cultivars in the Ecuadorian Andes: A phylogenetic and functional perspective
Source: PLoS One. 2026 Jan 2;21(1):e0339774. doi: 10.1371/journal.pone.0339774 (PMC12758762; doi:10.1371/journal.pone.0339774)
Supplement: Attachment — Submitted filename: 1_Rebuttal_letter_UCE_RhizobiumPlos_One.docx [file pone.0339774.s008.docx]

**Journal:** PLOS One

**ID manuscript:** PONE-D-25-33605

**Title:** Updating the description of Rhizobium diversity associated with common bean cultivars in the Ecuadorian Andes: a phylogenetic and functional perspective.

**Authors:** León-Cadena et al.

We would like to express our sincere gratitude to the Academic Editor and to the reviewers for their thorough evaluation of our manuscript and for their thoughtful, constructive comments. Their feedback has been invaluable for refining the manuscript and for improving its clarity and rigor. We have carefully considered all points raised and provide below a detailed, point-by-point response together with the corresponding revisions made in the manuscript.

**EDITOR**

**Comment 1:** Please ensure that your manuscript meets PLOS ONE's style requirements, including those for file naming. The PLOS ONE style templates can be found at

[https://journals.plos.org/plosone/s/file?id=wjVg/PLOSOne_formatting_sample_main_body.pdf](https://journals.plos.org/plosone/s/file?id=wjVg/PLOSOne_formatting_sample_main_body.pdf%20) and

<https://journals.plos.org/plosone/s/file?id=ba62/PLOSOne_formatting_sample_title_authors_affiliations.pdf>

**Response 1:** We thank the Editor for this comment. We have reviewed the PLOS ONE style guidelines and have revised the manuscript and all associated files to comply with the journal’s formatting and file-naming requirements. Specifically, we reformatted the main text according to the provided template, updated figure and table captions, and standardized file names to match PLOS ONE conventions. These changes are reflected in the revised submission.

**Comment 2:** In your Methods section, please provide additional information regarding the permits you obtained for the work. Please ensure you have included the full name of the authority that approved the field site access and, if no permits were required, a brief statement explaining why.

**Response 2:** We thank the Editor for this request. We have revised the Methods (Sampling subsection) to include the permit information and the approving authority. The inserted text now reads: “*Root nodules of P. vulgaris were collected systematically from agricultural soils across the Ecuadorian provinces mentioned, over the period 2019 to 2021. Sampling was conducted at 20 different locations, under research permits No. MAE-DNB-CM-2015-0028, No. MAE-DNB-CM-2015-0028-M-0001 and No. MAE-DNB-CM-2015-0028-M-003, issued by the Ministerio del Ambiente del Ecuador. Geographic coordinates for each site were recorded in Fig 1 and S1 Table”.* This change appears in the Methods section (Sampling subsection) of the revised manuscript.

**Comment 3:** We note that the grant information you provided in the ‘Funding Information’ and ‘Financial Disclosure’ sections do not match. When you resubmit, please ensure that you provide the correct grant numbers for the awards you received for your study in the ‘Funding Information’ section.

**Response 3:** We thank the Editor for highlighting this inconsistency. In accordance with the Editor’s instructions, we have removed all funding-related text from the manuscript so that funding information will appears only in the Funding Statement/online submission fields. The Funding Statement that we will provide in the cover letter and request the Editorial Office to enter in the online submission form is as follows:*“This study was supported by the Universidad Central del Ecuador through the Convocatoria de Proyectos de Investigación Senior UCE-2019 (DI-CONV-2019-033). The funders had no role in study design, data collection and analysis, decision to publish, or preparation of the manuscript.”*

At the time of resubmission we will verify that the Funding Statement in the cover letter, the Funding Information field in the online submission form, and the Financial Disclosure entry are identical.

**Comment 4:** Thank you for stating the following in the Acknowledgments Section of your manuscript: [This study was supported by the Universidad Central del Ecuador through the Convocatoria de Proyectos de Investigación Senior UCE-2019 (DI-CONV-2019-033). Additional support in logistics and laboratory infrastructure was provided by the Instituto de Investigación en Etnociencias and the Instituto de Investigación en Zoonosis (CIZ).]

We note that you have provided funding information that is not currently declared in your Funding Statement. However, funding information should not appear in the Acknowledgments section or other areas of your manuscript. We will only publish funding information present in the Funding Statement section of the online submission form.

Please remove any funding-related text from the manuscript and let us know how you would like to update your Funding Statement. Currently, your Funding Statement reads as follows: [This study was supported by the Universidad Central del Ecuador through the Convocatoria de Proyectos de Investigación Senior UCE-2019 (DI-CONV-2019-033). Additional support in logistics and laboratory infrastructure was provided by the Instituto de Investigación en Etnociencias and the Instituto de Investigación en Zoonosis (CIZ). The funders had no role in study design, data collection and analysis, decision to publish, or preparation of the manuscript].

Please include your amended statements within your cover letter; we will change the online submission form on your behalf.

**Response 4:** We thank the Editor for this clarification. We have removed the funding-related text from the Acknowledgments and retained only non-funding acknowledgement. The revised Acknowledgments now read:

**“Acknowledgements**

The authors are grateful for the logistical and laboratory support provided by the Instituto de Investigación en Etnociencias and the Instituto de Investigación en Zoonosis (CIZ).”

As noted above, the full Funding Statement (quoted in Response 3) will be provided in our cover letter and we respectfully request that the Editorial Office update the Funding Information in the online submission form to match that statement. We will confirm that the Funding Statement and the Financial Disclosure are identical before final submission.

**Comment 5:** Please amend either the abstract on the online submission form (via Edit Submission) or the abstract in the manuscript so that they are identical.

**Response 5:** We thank the Editor for this request. We will update the abstract entered in the online submission form (via Edit Submission) so that it exactly matches the abstract in the revised manuscript.

**Comment 6:** We note that you have included the phrase “data not shown” in your manuscript. Unfortunately, this does not meet our data sharing requirements. PLOS does not permit references to inaccessible data. We require that authors provide all relevant data within the paper, Supporting Information files, or in an acceptable, public repository. Please add a citation to support this phrase or upload the data that corresponds with these findings to a stable repository (such as Fig share or Dryad) and provide and URLs, DOIs, or accession numbers that may be used to access these data. Or, if the data are not a core part of the research being presented in your study, we ask that you remove the phrase that refers to these data.

**Response 6:** We thank the Editor for this important clarification. We have removed the phrase “data not shown” from the manuscript and have made the relevant data available as Supporting Information. Specifically, the material previously referenced as “data not shown” is now provided in **S1 Figure** (Supporting Information). As a result, the former S2 Figure has been renumbered accordingly. The manuscript text has been revised to cite the Supporting Information; the sentence now reads: *“Nucleotide similarity among the sequences, as well as with reference type strains, ranged from 82.4% to 100% (S1 Figure).”* All supporting files have been uploaded with the revised submission.

**Comment 7:** We note that Figure 1 in your submission contain [map/satellite] images which may be copyrighted. All PLOS content is published under the Creative Commons Attribution License (CC BY 4.0), which means that the manuscript, images, and Supporting Information files will be freely available online, and any third party is permitted to access, download, copy, distribute, and use these materials in any way, even commercially, with proper attribution. For these reasons, we cannot publish previously copyrighted maps or satellite images created using proprietary data, such as Google software (Google Maps, Street View, and Earth). For more information, see our copyright guidelines:

http://journals.plos.org/plosone/s/licenses-and-copyright.

We require you to either (1) present written permission from the copyright holder to publish these figures specifically under the CC BY 4.0 license, or (2) remove the figures from your submission:

1. You may seek permission from the original copyright holder of Figure 1 to publish the content specifically under the CC BY 4.0 license.

We recommend that you contact the original copyright holder with the Content Permission Form (http://journals.plos.org/plosone/s/file?id=7c09/content-permission-form.pdf) and the following text:

“I request permission for the open-access journal PLOS ONE to publish XXX under the Creative Commons Attribution License (CCAL) CC BY 4.0

(http://creativecommons.org/licenses/by/4.0/). Please be aware that this license allows unrestricted use and distribution, even commercially, by third parties. Please reply and provide explicit written permission to publish XXX under a CC BY license and complete the attached form.”

Please upload the completed Content Permission Form or other proof of granted permissions as an ""Other"" file with your submission.

In the figure caption of the copyrighted figure, please include the following text: “Reprinted from [ref] under a CC BY license, with permission from [name of publisher], original copyright [original copyright year].”

2. If you are unable to obtain permission from the original copyright holder to publish these figures under the CC BY 4.0 license or if the copyright holder’s requirements are incompatible with the CC BY 4.0 license, please either i) remove the figure or ii) supply a replacement figure that complies with the CC BY 4.0 license. Please check copyright information on all replacement figures and update the figure caption with source information. If applicable, please specify in the figure caption text when a figure is similar but not identical to the original image and is therefore for illustrative purposes only.

The following resources for replacing copyrighted map figures may be helpful:

USGS National Map Viewer (public domain): http://viewer.nationalmap.gov/viewer/

The Gateway to Astronaut Photography of Earth (public domain):

http://eol.jsc.nasa.gov/sseop/clickmap/

Maps at the CIA (public domain): https://www.cia.gov/library/publications/the-world-factbook/index.html and https:

//www.cia.gov/library/publications/cia-maps-publications/index.html

NASA Earth Observatory (public domain): http://earthobservatory.nasa.gov/

Landsat: http://landsat.visibleearth.nasa.gov/

USGS EROS (Earth Resources Observatory and Science (EROS) Center) (public domain): http://eros.usgs.gov/#

Natural Earth (public domain): <http://www.naturalearthdata.com/>

**Response 7:** Thank you for your comment. We have fully replaced Figure 1 with a new version created entirely by the authors using only public-domain geographic data. The map no longer uses mapview tiles, Google products, OpenStreetMap tiles, or any copyrighted basemaps. Instead, all boundaries come from Natural Earth (public domain), elevation data from openly accessible DEM sources obtained through the elevatr package, and all visual elements were rendered locally in R using sf, raster, and ggplot2. The figure caption has been updated accordingly. Because the revised figure contains only author-generated elements and public-domain datasets, it fully complies with the CC BY 4.0 license. The new Fig 1 caption reads as follows: “**Fig 1.** Map of Ecuador indicating the geographic locations of sampling sites in Imbabura (I), Pichincha (P), Chimborazo (Ch), and Loja (L) Provinces. Further details regarding these sampling areas are provided in S1 Table. “Basemap and administrative boundary data from Natural Earth (public domain). Elevation data from open-access DEM sources accessed via the elevatr package. Figure created entirely by the authors in R using sf, raster, and ggplot2 [30]. These data are compatible with publication under the CC BY 4.0 license from PLOS ONE.

"

**Comment 8:** Please include captions for your Supporting Information files at the end of your manuscript, and update any in-text citations to match accordingly. Please see our Supporting Information guidelines for more information: <http://journals.plos.org/plosone/s/supporting-information>.

**Response 8:** We thank the Editor for this reminder. We have now included detailed captions for all Supporting Information figures at Supporting Information section, and we have updated the in-text citations to ensure consistency with the revised captions. The text reads as follow:

**S1 Table. Geographic locations, coordinates and soil type of sampling sites for Rhizobium isolates.** This table provides the latitude, longitude, altitude, and soil type for each of the 46 collection sites used in this study.

**S2 Table. Growth tolerance of *Rhizobium* isolates to NaCl and temperature, and NCBI GenBank accession numbers for *16S rRNA*, *recA*, *glnII*, and *dnaK* sequences.** This table reports each isolate’s growth tolerance at the tested NaCl (1%, 2%) (w/v) concentrations and incubation temperatures (37 °C, 40°C), and lists the corresponding NCBI GenBank accession numbers for *16S rRNA*, *recA*, *glnII*, and *dnaK*.

**S3 Table. Primer sequences, PCR reaction components, and thermal cycling conditions used for amplification of target genes.** Lists primer names and sequences, reagent concentrations/volumes per PCR reaction, and the cycling parameters used for each target gene.

**S4 Table. Nodulation performance of two common bean (Phaseolus vulgaris) varieties (Canario [Ca] and Centenario [Ce]) inoculated with 43 rhizobial isolates and two reference strains under greenhouse conditions.** For each isolate–variety combination, the table reports the mean number of nodules (µ) and corresponding standard deviation (σ), based on three independent biological replicates. Nodules were scored 45 days post–inoculation. Isolates from Chimborazo were excluded due to unavailability at the moment of the greenhouse trial. In addition, an Excel (.xlsx) file will be included containing the mean (µ) and standard deviation (σ) values for each of the three independent biological replicates for the following variables: count of nodules > 2 mm, number of leghemoglobin–positive nodules, and percentage (%) of leghemoglobin-positive nodules. In yellow, isolates molecularly characterized with three housekeeping genes *recA*, *glnII* and *dnaK*.

**S5 Table. Raw data of the quantitative variables of nodulation performance evaluated in two varieties of bush bean Centenario (Ce) and Canario (Ca) after being inoculated with rhizobia isolates.** Raw data from the three replications of the three independent biological replicates for the following variables: i) number of nodules, ii) count of nodules > 2 mm, iii) number of leghemoglobin–positive nodules, and iv) percentage (%) of leghemoglobin-positive nodules.

**S1 Figure.** **Evolutionary divergence of *recA* sequences from study isolates and their closest described type strains.** Pairwise percent-identity values estimates were calculated from a 395 bp alignment of *recA* sequences to quantify the evolutionary divergence between each study isolate and its nearest described type strain. Evolutionary analyses were carried out using MEGA X; alignment and analysis parameters are detailed in the Methods.

**S2 Figure.**  **Evolutionary divergence of concatenated *recA*–*glnII*–*dnaK* sequences from study isolates and their closest described type strains.**

Pairwise percent-identity estimates were calculated from a 1,115 bp concatenated alignment (*recA*, *glnII*, *dnaK*) to quantify evolutionary divergence between each study isolate and its nearest described type strain. Evolutionary analyses were performed using MEGA X software; alignment and analysis parameters are described in the Methods.

**Comment 9:** If the reviewer comments include a recommendation to cite specific previously published works, please review and evaluate these publications to determine whether they are relevant and should be cited. There is no requirement to cite these works unless the editor has indicated otherwise.

**Response 9:** We thank the Editor for this guidance. Neither the reviewers nor the Editor recommended citing specific previously published works; consequently, no additional citations have been incorporated into the manuscript.

**Comment 10:** Please review your reference list to ensure that it is complete and correct. If you have cited papers that have been retracted, please include the rationale for doing so in the manuscript text, or remove these references and replace them with relevant current references. Any changes to the reference list should be mentioned in the rebuttal letter that accompanies your revised manuscript. If you need to cite a retracted article, indicate the article’s retracted status in the References list and also include a citation and full reference for the retraction notice.

**Response 10:** We thank the Editor for this important reminder. All 62 references listed have been **reviewed for any formal retraction notices, expressions of concern, or published corrections (errata)**. This review was done via a certified database and publisher records (leveraging Crossref’s Retraction Watch integration and PubMed/official journal updates). **None** of the references appear to have any formal retraction, expression of concern, or correction associated with them as of the current date. The key taxonomic and genome papers (e.g., Ribeiro et al. 2015 IJSEM describing Rhizobium ecuadorense and the 2015 genome announcement) appear on the publisher / PubMed sites with no retraction flags.

**ADDITIONAL MINOR CORRECTIONS**

**Administrative correction, author affiliation:** In the revised manuscript we have removed the outdated affiliation numbered **11** — Facultad de Ciencias Exactas Naturales y Ambientales (FCENA), Pontificia Universidad Católica del Ecuador, Quito, Ecuador — which was originally listed for Dr. Naranjo on the title page (lines 25–26 of the original submission). All other affiliations for Dr. Naranjo and for the co-authors remain unchanged from the original submission. This is a purely administrative correction and does not affect authorship order, author contributions, the data, or the conclusions of the manuscript. Dr. Naranjo has reviewed and approved the revised version.

**Numerical and dataset adjustments:** We identified and corrected numerical transcription errors in Table 1 (pairwise nucleotide similarity ranges; see also Supplementary Figure S2). In addition, we removed from Cluster C3, in Table 1, two previously listed reference strains (R. sophoriradicis and R. esperanzae), which are not present in Fig. 3; consequently, only R. phaseoli ATCC 14482ᵀ and R. etli CFN 42ᵀ remain as reference strains for the MLSA comparison of cluster C3. The corrected version now reads as follows:

| **Cluster  name** | **Sub-cluster  name** | **UCE  strains** | **Nucleotide similarities among local isolates  (range %)** | **Range of nucleotide similarities  with the closest type strain  (%)** |
| --- | --- | --- | --- | --- |
|  |  |  |  |  |
| C1 | C1.1 |  | 99.8%–100% | *R. ecuadorense CNPSo 671ᵀ*  (98.8%–98.9%) *R. leguminosarum* bv. viciae USDA 2370ᵀ (96.6%–96.7%) |
|  |  | **UCE**0001 P |  |  |
|  |  | **UCE**0043 I |  |  |
|  |  | **UCE**0080 I |  |  |
|  |  | **UCE**0082 I |  |  |
|  |  | **UCE**0117 I |  |  |
|  |  | **UCE**0148 L |  |  |
|  |  | **UCE**0221 Ch |  |  |
|  |  |  |  |  |
|  | C1.2 |  | 96.5%–99.2% | *R. ecuadorense* CNPSo 671ᵀ  (96.2%–97.8%) *R. leguminosarum* bv. viciae USDA 2370ᵀ (95.1%–95.7%) |
|  |  | **UCE**0024 I |  |  |
|  |  | **UCE**0027 I |  |  |
|  |  | **UCE**0042 I |  |  |
|  |  | **UCE**0060 I |  |  |
|  |  | **UCE**0203 I |  |  |
|  |  |  |  |  |
| C2 | - |  | 100% | *R. pisi* DSM 30132ᵀ (92.9%) |
|  |  | **UCE**0224 Ch |  |  |
|  |  | **UCE**0228 Ch |  |  |
|  |  |  |  |  |
| C3 | - |  | 95.2%. | *R. phaseoli* ATCC 14482ᵀ  (94.9%–99.0%) *R. etli* CFN 42ᵀ (93.3%–91.6%) |
|  |  |  |  |  |
|  |  |  |  |  |
|  |  | **UCE**0055 I |  |  |
|  |  | **UCE**0231 Ch |  |  |
|  |  |  |  |  |
|  |  |  |  |  |
|  |  |  |  |  |
| C4 | - |  | 97.6%–100% | *R. tropici* CIAT 899ᵀ (94.1%–94.9%) *R. jaguaris* CCGE525ᵀ (89.6%–90.2%) |
|  |  | **UCE**0158.2 L |  |  |
|  |  | **UCE**0150 L |  |  |
|  |  | **UCE**0158 L |  |  |
|  |  |  |  |  |

Based on these corrections, we modified the corresponding paragraphs in the Results section. The revised paragraphs now read as follows:

**1)** “Within C1.2, isolates showed nucleotide similarities of 96.5% to 99.2% with each other, from 96.2% to 97.8% with *R. ecuadorense* CNPSo 671ᵀ, and from 95.1% to 95.7% with *R. leguminosarum* bv. viciae USDA 2370ᵀ.”

**2) “**Cluster C3 comprised isolates UCE0055 and UCE0231, which showed a pairwise nucleotide similarity of 95.2% with each other. Comparative analysis with reference strains indicated that these isolates shared nucleotide similarities of 94.9%−99.0% with R. phaseoli ATCC 14482ᵀ and 93.3%−91.6% with R. etli CFN 42ᵀ.”

Accordingly, we also updated the Discussion paragraph that refers to these results. The corrected sentence now reads:

**1)** In contrast, subcluster C1.2 exhibited 96.5–99.2% sequence similarity among isolates, but <97.8% similarity to its closest type strains, *R. ecuadorense* CNPSo 671*ᵀ* and R. leguminosarum USDA 2370ᵀ.

Finally, we removed strain UCE0074 in C3 cluster (original manuscript, line 359) and excluded the two previously listed reference strains *R. sophoriradicis* and *R. esperanzae*. The removal of UCE0074 reflects an earlier decision recorded during sample processing, to omit this isolate because no nodulation data were obtained; its inclusion in the original submitted draft was an inadvertent oversight (it appeared in the text but not in the table). We have updated the similarity ranges for clusters C1.2 and C3 and the corresponding reference-strain ranges accordingly (see revised Table 1). We rechecked the MLSA/distance outputs after these edits and confirm that these are data-correction edits only and do not affect the clustering topology, statistical analyses, or the conclusions of the manuscript. The revised Table 1 is included in the resubmission, and all changes are visible in the marked-up file.

**Orthography corrections (editorial clarification):** We made minor orthographic and grammatical corrections throughout the manuscript to improve clarity, consistency, and adherence to academic English standards. These edits involved the correction of typographical errors, verb forms, punctuation, and article use, without altering the meaning, data interpretation, or conclusions of the study.

1) Despite its ecological significance, comprehensive analyses of rhizobial diversity remain limited, particularly in the Ecuador-Peru region, a recognized genetic reservoir for *P. vulgaris* and one of its primary centers of diversification, where coevolutionary dynamics between *P. vulgaris* and its microsymbionts have shaped their speciation and distribution…
2) The isolates grouped in cluster C3, UCE0055, and UCE0231 were positioned within the R. etli/R. phaseoli complex. UCE0055 and UCE0231 showed nucleotide similarities…
3) Notably, under controlled greenhouse conditions, the most effective isolates belonged to C1 cluster…
4) Therefore field trails are require to validate the nodulation competitiveness and symbiotic effectiveness of these elite native strains under agronomic conditions…
5) Notably, under controlled greenhouse conditions, the most effective isolates belong to C1 cluster (closely related to *R. ecuadorense*) and C4 cluster (affiliated with *R. tropici*) **with varietal-specific dominance patterns** (Tables 2 and S4). Therefore, field trials are required to validate the nodulation competitiveness and symbiotic effectiveness of these elite native strains under agronomic conditions, **and to** sequence nodC genes **to test the hypothesis of Aguilar et al.**
6) Colonies were subcultured in YMA plus 0.5% of bromothymol blue (BTB) indicator to assess acid/alkali production and subsequently on YMA to obtain pure cultures.
7) Briefly: the nodules, placed inside a sterile tea strainer, were successively submerged in 70% v/v ethanol for 1 min…
8) Individual colonies from each isolate were subsequently re-plated on fresh YMA plates.
9) Rhizobia strains were grown on agar plates for 2–5 days, suspended in sterile deionized water, and adjusted to a final concentration of 10^9^ CFU mL^-1^.
10) Amplification and sequencing of 16S rRNA gene and housekeeping genes *recA, glnII* and *dnaK****.***
11) High-purity DNA was stored at -20 °C and subsequently used as a template for PCR amplification of the *16S rRNA*, *recA*, *glnII* and *dnaK* genes
12) Bootstrap values (1000 replicates) above 70% are represented at the nodes.
13) Isolates from Chimborazo province (UCE0221, UCE0224, UCE0228, and UCE0231) were collected after the COVID-19 pandemic due to mobility constraints and following the completion of the greenhouse trials.
14) The nodulation capacity was evaluated by inoculating of 42 of 46 rhizobia isolates…
15) The ribosomal RNA (rRNA) gene was successfully amplified in 16 of 19 plus two representative strains.
16) Our results suggest geographic variation among Ecuadorian *Rhizobium* populations and identify locally predominant groups that could be evaluated further as bioinoculants for bean cultivation.
17) The phylogenetic tree constructed from these sequences grouped the isolates into nine distinct clusters: R1 (26 isolates, 56.5%), R2 (1 isolate, 2.2%), R3 (2 isolates, 4.3%), R4 (1 isolate, 2.2%), R5 (1 isolate, 2.2%), R6 (3 isolates, 6.5%), R7 (4 isolates, 8.7%), R8 (2 isolates, 4.3%) and R9 (6 isolates, 13.0%)…

**Specification of concentration units (w/v) (editorial clarification):** We identified that the concentration units were not previously specified in some methodological descriptions. To improve clarity and precision, we added the indication “(w/v)” where appropriate. The corrected sentences now read as follows:

i) “Furthermore, tolerance to NaCl (1% and 2%) (w/v) was evaluated on YMA at 28 °C, and growth was also evaluated at two different temperatures (37 °C and 40 °C) on YMA…”
ii) “The quality and concentration of purified DNA were determined using a NanoDrop 2000 spectrophotometer (Thermo Scientific, Waltham, USA) and 1% (w/v) agarose gel. High-purity DNA was stored at -20 °C and subsequently used as a template for PCR amplification.”

We noted a minor inconsistency in the way the ClustalW alignment software was cited in two sections of the manuscript. To maintain uniformity in style and nomenclature, we standardized both instances to refer to ClustalW. The corrected sentences now read as follows:

i) “Both sets of sequences were exported and aligned using ClustalW within MEGA X [35].”
ii) “These housekeeping genes were aligned using ClustalW in MEGA X and appropriately trimmed.”

**Abbreviation of Multilocus Sequence Analysis to MLSA (editorial):** In the revised manuscript we removed the full phrase “Multilocus Sequence Analysis” in two instances and retained only the acronym “MLSA,” as the term had already been defined earlier in the text. The corrected sentences now read as follows:

i) “To further resolve the taxonomic position of these isolates, we performed a 16S rRNA sequence analysis followed by MLSA using concatenated sequences of the housekeeping genes recA, glnII, and dnaK.”
ii) “Foundational studies identified R. ecuadorense as a novel nitrogen-fixing symbiont phylogenetically positioned within the R. phaseoli/R. etli/R. leguminosarum clade but genomically and phenotypically different, justifying its classification as a novel species, exhibiting <94.2% nucleotide identity in MLSA and…”

**Correction to Materials and Methods (units):** We identified that the dimensions of the polyethylene bags in the original manuscript were reported in inches and have converted these to the International System of Units (SI). The Methods text has been updated accordingly. The revised sentence now reads: “Pre-germinated bean seeds were individually transplanted into **38.1 cm × 50.8 cm** free-draining polyethylene bags filled with 0.5 kg of steam-sterilized vermiculite as substrate.” This is an editorial unit conversion only and does not affect the experimental procedures, data, analyses, or conclusions.

**REVIEWERS**

**Reviewer 1**

**Comment 1:** The article has been significantly improved after revision and all my previous concerns have been addressed to my satisfaction. I recommend it for publication. I recommended an English revision, and it is possible to measure nitrogenase activity.

**Response**: We thank for the positive evaluation and for recommending the manuscript for publication. For clarification, this submission represents our first revised version following the initial peer review. However, we have performed a carefully English-language revision throughout the manuscript to improve clarity and readability. All the changes reviewed appears in the track changes manuscript in blue highlight. Measurement of nitrogenase activity (e.g., by the acetylene reduction assay) was not included in the present study and could not be performed due to resource limitations.

**REVIEWER 2**

**Comment 1***:* The manuscript contains valuable research and suitable methods (MLSA). However, several items require refinement or correction: (1) Figure 2 is not readable — image quality must be enhanced; (2) Figure 4 is blurred and not interpretable — visibility must be improved; (3) Table No. 4 (referred to in the text) is missing — please upload it so the variation in nodule number can be evaluated.

**Response 1***:* We appreciate Reviewer 2’s careful critique and have addressed each point as follows.

1. **Figure 2 (readability and quality):** We have replaced Figure 2 with a high-resolution version (500 dpi). The figure legend has also been expanded to be self-contained and to describe all symbols and annotations.
2. **Figure 4 (clarity and visibility):** Figure 4 has likewise been replaced with a high-resolution image (500 dpi) and reformatted to improve visibility

**Table 4 (missing):** We apologize for the omission. S4 Table is now available in the supplementary information and the description reads as follow: “**S4 Table. Nodulation performance of two common bean (Phaseolus vulgaris) varieties (Canario [Ca] and Centenario [Ce]) inoculated with 43 rhizobial isolates and two reference strains under greenhouse conditions.** For each isolate–variety combination, the table reports the mean number of nodules (µ) and corresponding standard deviation (σ), based on three independent biological replicates. Nodules were scored 45 days post–inoculation. Isolates from Chimborazo were excluded due to unavailability at the moment of the greenhouse trial.
